# Quinine Test

**Published:** 1880-02

**Authors:** 


QUININE TEST.—The following simple test has been proposed by Mr. O. Hesse in a recent issue of the *Archiv. der Pharmacie.* Agitate 0.5 gramme of the sulphate of quinine to be tested with 10 c.c. of water at 50° to 60° Celsius. After cooling filter and put 5 c.c. of the filtered solution in a bottle, cover with 1 c.c. ether, and add 5 drops of liquor ammonia. Cork the bottle, agitate lightly a few times, and set aside for two hours. If no crystals are observable in the ether layer, even by the aid of the microscope, the sulphate of quinine was pure. Small granular crystals prove the presence of cinchonidin, and needles in concentric groups indicate cinchonine, or cinchonin. C. Rump has since communicated to the *Pharmaceutische Zei-*

*tung* a comment on this test. He considers that the 0.5 gramme of sulphate of quinine should be put in a test tube, weigh on it 10 grammes of distilled water, hold the tube in hot water until the necessary temperature has been reached. After filtering as before, weigh 5 grammes in another tube, and to this weigh one gramme of ether, adding 3 to 5 drops of liquor ammonia. Set aside as in the other case. He considers 1 c.c. of ether too little.

Dr. C. Schacht, of Berlin, has communicated to the *Pharmaceutische Zeitung* the results of experiments made by him with this test. He records the following results: Sulphate of quinine manufactured by Bohringer (Manheim,) Koch (Oppenheim,) Chinin-Fabrik (Brunswick,) Conrad Zimmer (Frankfort-on-the-Main,) all yielded 3 per cent. of sulphate of cinchonidia and homo-cinchonidia. That manufactured by Jobst (Stuttgart,) was pure. Of foreign quinines he found Howard's pure, while Pelletier's contained 1 per cent., and a sample from Milan 3 per cent.—*Monthly Review Medicine and Pharmacy.*

